# The Impact of Work Demands and Meaningful Work on the Burnout and Mental Health Strain of Emergency Medicine Clinicians

**DOI:** 10.1002/smi.70160

**Published:** 2026-03-12

**Authors:** Alyssa Lynch, Thomas W. Britt, Marissa Shuffler, Riley McCallus, Emily Hirsh

**Affiliations:** ^1^ Department of Psychology Clemson University Clemson South Carolina USA; ^2^ Department of Psychology Furman University Greenville South Carolina USA; ^3^ Prisma Health Department of Emergency Medicine Greenville South Carolina USA; ^4^ University of South Carolina School of Medicine‐Greenville Greenville South Carolina USA

**Keywords:** burnout, COVID‐19, emergency medicine, healthcare, mental health

## Abstract

Emergency medicine clinicians (EMCs) experience high levels of burnout and mental health strain as a function of the work demands they encounter. The present study examined meaningful work as a buffer of the relationship between work demands and measures of burnout and mental health strain, with the prediction that higher levels of meaningful work would protect EMCs from high levels of work demands. This study offers a longitudinal examination of these relationships in a sample of 113 emergency medicine registered nurses, physicians, residents, and advanced practice providers during the COVID‐19 pandemic. Participants completed an online survey containing measures of work‐related demands, meaningful work, burnout, and mental health strain at Time 1 and then completed the same survey 2 months later at Time 2. Work demands at Time 1 were correlated with burnout and mental health strain at Time 2. Meaningful work at Time 1 correlated negatively with burnout and mental health strain at Time 2. Moderated multiple regression tests revealed that meaningful work at Time 1 moderated the relationship between work demands at Time 1 and burnout at Time 2, such that the relationship between work demands and burnout was not significant for EMCs reporting average or high meaningful work. Moderate to high levels of meaningful work at Time 1 substantially reduced the impact of work demands on EMCs 2 months later. Results suggest that interventions should be explored to enhance meaningful work, thereby reducing the negative effects of work demands on burnout and mental health strain.

## 1 Introduction

The subject of healthcare worker burnout and well‐being has been an important issue, especially within emergency medicine. Studies have shown that 65%–74% of residents across all specialties meet the criteria for burnout, revealing that burnout starts even in the early stages of professional development (Takayesu et al. 2014; Shanafelt and et al. 2012). The problem of burnout and mental health among healthcare professionals is so prevalent that the National Academies of Sciences and et al. (2019) have developed a systematic effort to address burnout and enhance the well‐being of providers. Although burnout is prevalent across healthcare specialties, the Medscape Physician Burnout and Depression Report 2024 revealed that emergency medicine ranked at the top of the list of feeling burnout at a rate of 63% (McKenna 2024). Emergency medicine clinicians (EMCs) experience unique stressors that can contribute to their elevated levels of burnout and work stress and that negatively affect their physical, emotional, and mental health. However, EMCs also often find meaning in their work as healthcare providers, which could provide these clinicians with an important resource to blunt the negative impact of work stressors (Allan et al. 2016).

The present study examined predictors of burnout and mental health strain among EMCs, addressing general work demands and work demands related to the COVID‐19 pandemic that these healthcare workers faced. Additionally, this study examined how meaningful work could be a buffer against work demands, lessening the effects on burnout and negative mental health strain. At a time at which the healthcare industry is examining ways to reduce burnout and mental health strain, this study highlights meaningful work as a factor that could lessen these responses to work demands.

In the present study, EMCs at an academic medical centre (nurses, physicians, and advanced practice providers) were assessed at two different time periods during the COVID‐19 pandemic to examine how work‐related demands and perceptions of meaningful work at Time 1 were related to burnout and mental health strain two months later at Time 2. The short‐term reaction model of how stressors are related to strains was utilised in the present study, where stressors are hypothesised to have an immediate direct effect on the strain, with reactions only diminishing shortly if there is no longer exposure to the stressors (Garst et al. 2000). In the remainder of the Introduction, we provide background research on burnout and mental health strain, unique demands of emergency medicine, and the hypotheses of the present research.

Burnout is a psychological syndrome that consists of emotional exhaustion, depersonalisation, and reduced personal accomplishment, a commonly seen issue in healthcare (Maslach and Leiter 2016). Emotional exhaustion consists of the feeling of being “used up” at the end of the working day and not being able to emotionally support patients (West et al. 2018). Depersonalisation includes dehumanising patients and treating them as objects, again emotionally disconnecting and seeming uncaring. Reduced personal accomplishment is seen as feeling ineffective when caring for patients' problems and feeling a lack of value in patient care or professional achievements. Maslach and Leiter (2016) discovered that these elements primarily manifest in professions where individuals work closely with others, such as healthcare.

The present study used the Job Demands‐Resources Model (JDR) of Bakker and Demerouti (2017) to understand the predictors of burnout among healthcare professionals, as well as the potential beneficial effects of meaningful work on reducing burnout. According to the JD‐R model, burnout is the result of work demands that are encountered in the occupational setting, as well as the job resources that are available to employees for addressing the demands that are present. Emergency medicine brings unique demands compared to other specialties, and it is important to recognise the effects of these demands on clinicians. Moukarzel et al. (2019) discovered that EMCs excessive workload and high care demand contribute to higher burnout levels. Portero de la Cruz and et al. (2020) examined nurses working in the emergency department and found that higher levels of burnout were associated with perceived stress in the emergency department due to lack of personnel, work overload, shift work, role ambiguity, lack of autonomy, rapid technological changes, and increased pressure in decision‐making.

EMCs also face further work demands that include a need to be knowledgeable of a wide range of medical, surgical, and psychological problems, not only critical illnesses and injuries (Ayatollahi and et al. 2015). The patients EMCs oversee are unpredictable, leading for the need to be prepared for any type of case. EMCs must provide immediate care for patients with undifferentiated and often life‐threatening conditions, focussing on rapid diagnoses and stabilisation. This contrasts with other specialties that typically deal with established diagnoses and require long‐term care for chronic conditions (Ayatollahi and et al. 2015). The emotional and mental strain of the job is compounded by situations involving patients with suicidal behaviours or those without access to timely psychiatric care, adding another layer of stress (Castner 2019). EMCs also work more unpredictable hours than other specialists due to the 24/7 nature of emergency care, leading to irregular sleep patterns and disrupted circadian rhythms that contribute to both physical and mental fatigue (Berger 2013).

Along with these demands, emergency departments also commonly face workplace violence, with 91% of emergency physicians reporting being threatened or attacked in the past year, according to the 2024 American College of Emergency Physicians poll, creating the additional layer of stress and concern for personal safety. Research has shown that emergency physicians also experience more interruptions and manage more patients than primary care physicians (Chisholm et al. 2001).

In addition to traditional work demands as predictors of burnout among healthcare professionals, the COVID‐19 pandemic produced additional demands that exacerbated the problem of burnout and well‐being. Ceri and Cicek (2021) found that healthcare professionals who worked on the frontlines during COVID‐19 experienced greater stress and worsened mental health. These effects were hypothesised to be a function of additional pandemic‐related stressors brought to the healthcare work environment, including cases of COVID‐19 rapidly increasing while cases not suspected to be due to COVID‐19 dropped below the standard in multiple emergency departments (Comelli and et al. 2020). This led to an unexpected patient pattern shift, resulting in a need for quick implementation of organizational processes and even new triage criteria. Personal protective equipment (PPE) was essential for healthcare workers' safety, but the increased demand resulted in having to ration its use, leaving workers to continue to worry about their safety and susceptibility to being infected with COVID‐19 (Ferrer 2020).

According to the JD‐R model, job demands represent those factors in the work environment that require continuous physical, mental, or emotional effort. EMCs face numerous job demands that are consistently present in their work environment. In addition, the COVID‐19 pandemic brought additional job demands that added to the overall level of work stressors facing EMCs. In the present study we developed a checklist of job demands that included general work demands in the clinical environment and work demands that were brought about as a result of the COVID‐19 pandemic (Brooks et al. 2018; Maunder et al. 2006; Tam et al. 2004).

According to the JD‐R model, job resources are directly related to lower levels of burnout and can also partially offset the work demands facing employees in a given context. Job resources refer to those factors in the work environment that facilitate achievement of work‐related goals, help reduce job demands, or stimulate professional growth (Bakker and Demerouti 2007). Resources can also come from various aspects of the work environment, including physical (e.g., ventilators to help address lung functioning), psychological (e.g., feeling autonomy at work), social (e.g., supervisor support), or organizational (e.g., information sharing by leadership).

In the present study we argue that finding meaningfulness in work represents an important job resource that can serve to buffer healthcare workers from work demands and help them counter burnout. Meaningful work can be considered a psychological state where individuals feel they make a positive and valuable contribution to a worthwhile purpose, such as having a beneficial impact on clients' lives (Yeoman 2014). Martela and Pessi (2018) argued that meaningful work is driven by the significance and purpose of work. Work significance indicates that work is intrinsically valuable and worth doing; a broader purpose is defined as work serving some greater good beyond the individual. Meaning and purpose are also synergistic components for sustained engagement and creativity among clinicians and are critical to their professional identities, as clinicians have emphasised the importance of finding meaning in their work (Tak et al. 2017; Rushton 2018).

Some prior research has found that meaning derived from work is an important job resource for emergency physicians. One study with Israeli emergency physicians discovered that meaning derived from work was an influential factor in work satisfaction and burnout, especially in the later stages of emergency physicians' careers (Ben‐Itzhak et al. 2015). Another study found that nurses who reported a “calling to nursing” experienced higher meaningfulness in work, career commitment, personal well‐being and satisfaction, and work engagement (Ziedelis 2019). This feeling of calling might be a source of motivation for healthcare personnel to engage in their work despite the demanding work environment. According to Park's (2013) meaning‐making model, meaningful work should moderate the negative relationship between work stress and negative outcomes, buffering employees from the negative effects of work‐related stressors.

Based on the JD‐R model and prior research examining work demands, meaningful work, and burnout among healthcare providers, the following hypotheses were proposed.

Hypothesis 1 Higher levels of work demands at Time 1 will be associated with higher burnout and mental health strain 2 months later at Time 2.

Hypothesis 2 Higher levels of meaningful work at Time 1 will be associated with lower burnout and mental health strain 2 months later at Time 2.

Hypothesis 3 Meaningful work at Time 1 will moderate the relationship between work demands at Time 1 and burnout at Time 2, and between work demands at Time 1 and mental health strain at Time 2. The relationships between the demands and indicators of strain are expected to be lower for those clinicians reporting high levels of meaningful work.

## 2 Method

This study used a longitudinal survey design to examine job demands, meaningful work, burnout, and mental health strain among EMCs during a high‐stress period of the COVID‐19 pandemic. Key elements of the data collection and participants are provided in Table 1. Data collection occurred at two timepoints, July 2021 and September 2021, corresponding with the Delta variant surge and a national push for vaccination (Centers for Disease Control and Prevention 2021). EMCs were clearly in a heightened period of work demands and at risk for burnout and mental health problems during this time frame. Participants worked at one or more of seven emergency departments across Upstate South Carolina. The following sections detail the study sample, measures used, and procedure for data collection.

**TABLE 1:** Overview of data collection and participants.

Assessment time frame	Total participants	Occupation	Gender
Time 1 (July 2021)	180	Registered nurse: 100 Attending physician: 53 Advanced practice provider: 20 Resident: 7	Female:124 Male: 55 No answer: 1
Time 2 (September 2021)	171	Registered nurse: 83 Attending physician: 60 Advanced practice provider: 17 Resident: 11	Female:109 Male: 61 No answer: 1

### 2.1 Participants

As seen in Table 1, the Time 1 assessment included 180 participants and the Time 2 assessment included 171 participants. The 113 participants who successfully completed both the July 2021 and September 2021 assessments were included in the present study. The majority of the sample consisted of Nurses, followed by Physicians, Advanced Practice Providers, and Residents. The majority of the sample was female.

### 2.2 Measures

#### 2.2.1 Job Demands

In developing a checklist of work demands for the present study, our research team conducted discussions with EMCs and also consulted prior research on the topic (Brooks et al. 2018; Maunder et al. 2006; Tam et al. 2004). The goal was to develop a checklist that would represent the key job demands of EMCs, including nurses and physicians. The checklist that was developed contained job demands that were both general and specific to the COVID‐19 pandemic. Clinical concerns in the work environment were assessed with six items. Examples include “staffing concerns in the hospital (e.g., too few doctors, too few nurses, too few staff),” “issues with supplies needed to treat patients (e.g., shortages, availability, organization),” and “lack of clarity around caring for suspected COVID‐19 patients (e.g., PPE, testing, treatment).” General work‐related concerns were assessed with eight items. Example items include “fear that my job is at risk,” “issues with scheduling (e.g., schedule turnaround, time off requests, fairness),” “keeping up with frequent changes,” “lack of support from the hospital/organization,” and “concern that my education/training is being negatively impacted by the pandemic.” These items were created for the present study but were also similar to demands assessed in previous studies in terms of content and the unique demands of the COVID‐19 pandemic (Castner 2019; González‐Gil et al. 2021). All items were combined into a single measure of work demands.

#### 2.2.2 Meaningful Work

Participants completed a Likert‐type scale developed for the present study consisting of five items addressing different aspects of meaningful work. These statements included feeling that their work was meaningful and impactful, as well as the positive outcomes of work. Participants were asked “How often have the following been true for you in the past month” and responded to the items on a five‐point scale anchored by Never/Hardly Ever and Always. Example items include “I felt that my work was meaningful” and “I positively impacted patients and their families.” Although the scale was developed for the present study, the items were consistent with measures of meaningful work used in other studies. For example, the item “I felt that my work was meaningful” is similar to the item “My job gives me meaning” used by Ben‐Itzhak et al. (2015). Additionally, the item “I positively impacted patients and their families” is consistent with Martela and Pessi's (2018) point that serving a broader purpose is a dimension of meaningful work. The measure of meaningful work had a Cronbach's Alpha of 0.87 in the present study at Time 1, showing high reliability.

#### 2.2.3 Burnout

Burnout was measured with the Mini Z Single Item Burnout Measure (Dolan et al. 2015). Participants were asked to pick a description that characterised their level of burnout. Sample options include “I enjoy my work, I have no symptoms of burnout”, “I am definitely burned out and have one or more symptoms of burnout'”, “I feel completely burned out”, and “I am at the point where I may need to seek help”. The Mini Z survey has been validated against the Maslach Burnout Inventory Emotional Exhaustion Subscale and performed similarly in identifying stressors associated with burnout (Dolan et al. 2015).

#### 2.2.4 Mental Health Strain

To measure mental health strain, a modified version of the Mayo Clinic Physician Well‐Being Index (PWBI) was used, consisting of six items (Dyrbye et al. 2014). Sample items include “Have you fallen asleep while stopped in traffic or driving?” and “Have you been bothered by emotional problems (such as feeling anxious, depressed, or irritable?”. Clinicians were instructed to pick either yes or no for each of the items. The burnout item was omitted due to the use of a different burnout measure being analysed separately. A previous study by Dyrbye et al. (2014) established the PWBI's construct validity. They found significant relationships between the PWBI and the variables of depression, burnout, fatigue, and mental quality of life when tested with a national sample of residents.

### 2.3 Procedure

The present study was part of a larger study that assessed a broader range of EMC outcomes during COVID‐19. Participants first took weekly surveys (March 25‐April 30, 2020; see Britt et al. 2021), then transitioned to monthly surveys throughout the COVID‐19 pandemic. Participants were sent personalised survey links to their work emails monthly, using a web‐based survey software tool (Qualtrics). The survey remained open for 6 days to include the weekend and several weekdays, minimising EMCs' barriers to completion caused by schedule variations and email access. Each survey contained questions asked in relation to the specific month that it was sent out, to prevent overlap between time frames. Respondents received a $5 gift card as compensation for completion. Only fully completed surveys were included in analysis. The survey‐based effort was originally designed to provide department leadership with regular feedback using brief assessments measuring the burnout and well‐being of emergency medicine professionals at an academic health centre in the Southeastern United States. Ethics approval for research use of the survey results was obtained through IRB approval from the academic health centre.

## 3 Results

### 3.1 Descriptive Statistics and Correlations Among the Measured Variables

Time 1 and Time 2 descriptive statistics among the measured variables are found in Table 2. In terms of differences between the key variables from Time 1 to Time 2, the average number of work demands increased from Time 1 to Time 2, *t*(112) = −3.98, *p* < 0.001. Burnout also increased from Time 1 to Time 2, *t*(112) = −5.79, *p* < 0.001, as did mental health strain, *t*(112) = −5.89, *p* < 0.001. In contrast, meaningful work decreased from Time 1 to Time 2, *t*(112) = 4.36, *p* < 0.001. The differences between the primary variables from Time 1 to Time 2 likely reflect the increased incidence of COVID‐19 cases from July to September of 2021 as a function of the COVID‐19 Delta variant.

**TABLE 2:** Means and standard deviations among the measured variables at time 1 and time 2.

Variable	Mean (SD) at time 1	Mean (SD) at time 2
Meaningful work	3.60 (0.75)	3.33 (0.73)
Work demands	4.51 (2.54)	5.66 (2.95)
Mental health strain	1.97 (1.82)	2.98 (1.95)
Burnout	2.31 (0.8)	2.7 (0.91)

The Pearson correlations among the continuous variables can be found in Table 3. Most of the correlations were moderate in strength, including between mental health strain and meaningful work at Time 1, mental health strain and work demands at Time 1, burnout and meaningful work at Time 1, and burnout and mental health strain at Time 1. Moderate correlations were also found between meaningful work at Time 1 and mental health strain and burnout at Time 2, and work demands at Time 1 and mental health strain and burnout at Time 2. In support of Hypothesis 1, level of work demands at Time 1 was significantly correlated with burnout and mental health strain at Time 2. In support of Hypothesis 2, levels of meaningful work at Time 1 were significantly correlated with burnout and mental health strain at Time 2.

**TABLE 3:** Correlations among the measured variables at time 1 and time 2.

Variable	Meaningful work (T1)	Work demands (T1)	Mental health strain (T1)	Burnout (T1)	Meaningful work (T2)	Work demands (T2)	Mental health strain (T2)	Burnout (T2)
Meaningful work (T1)	—							
Work demands (T1)	−0.44**	—						
Mental health strain (T1)	−0.52**	0.41**	—					
Burnout (T1)	−0.56**	0.48**	0.72**	—				
Meaningful work (T2)	0.68**	−0.31**	−0.39**	−0.55**	—			
Work demands (T2)	−0.21*	0.48**	0.28**	0.32**	−0.38**	—		
Mental health strain (T2)	−0.50**	0.40**	0.60**	0.54**	−0.56**	0.51**	—	
Burnout (T2)	−0.47**	0.33**	0.55**	0.75**	−0.56**	0.51**	0.75**	—

^*^ *p* < 0.05.

^**^ *p* < 0.01.

### 3.2 Meaningful Work as a Moderator of the Relationship Between Work Demands and Burnout and Mental Health Strain

**FIGURE 1:**
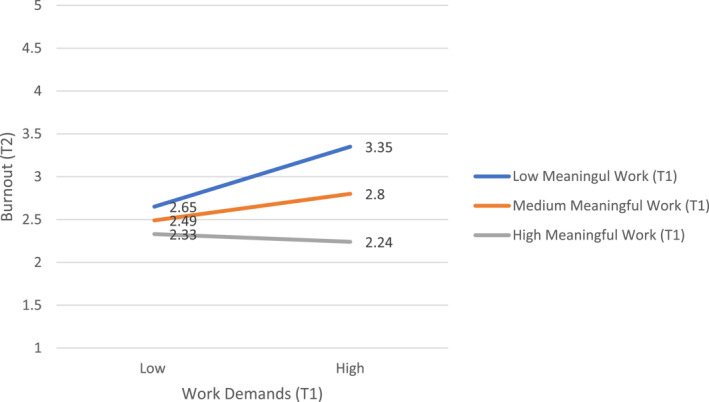
The interaction between meaningful work and work demands at time 1 as predictors of burnout at time 2.

Moderated multiple regressions were used to examine meaningful work as a moderator of relationships between work demands at Time 1 and the negative outcomes at Time 2. Work demands and meaningful work were mean‐centred prior to forming an interaction term between the two variables to test for moderation. The results of the moderated multiple regression tests can be found in Table 4. The analyses revealed a significant interaction between work demands and meaningful work at Time 1 predicting burnout at Time 2. The interaction between work demands and meaningful work at Time 1 was not a significant predictor of mental health strain at Time 2. The interaction of meaningful work and work demands at Time 1 as a predictor of burnout at Time two is described in Figure 1.

**TABLE 4:** Moderated mediation results for work demands (T1) and meaningful work at (T1) as predictors of burnout (T2) and mental health strain (T2).

	Burnout (T2)				Mental health strain (T2)			
Predictor	B	SE	B	*t*	B	SE	B	*t*
Work demands (T1)	0.060	0.033	0.159	1.840	0.200	0.069	0.248	2.895**
Meaningful work (T1)	−0.478	0.104	−0.396	−4.607**	−1.043	0.219	−0.406	−4.759**
Work demands X meaningful work (T1)	−0.104	0.038	−0.222	−2.749**	−0.058	0.079	−0.058	−0.728

*p < 0.05.

^**^ *p* < 0.01.

The pattern shown in Figure 1 supported a buffering effect, with the relationship between work demands at Time 1 and burnout at Time 2 being weaker for those clinicians reporting higher meaningful work at Time 1. More specifically, simple slope tests were conducted at low, medium, and high levels of meaningful work (−1, 0, and 1 standard deviations from the mean‐centred meaningful work variable). These tests revealed the relationship between work demands and burnout was significant for those reporting low meaningful work, *t*(108) = 3.69, *p* < 0.01, but was not significant for those reporting moderate, *t(*108) = 1.90, *p* = 0.06, or high, *t*(108) = −0.43, *p* = 0.67, meaningful work. Importantly, the interaction between meaningful work and work demands as a predictor of burnout at Time 2 remained significant when burnout at Time 1 was controlled, *t* = −2.71, *p* = 0.008. These findings partially support Hypothesis 3, as meaningful work at Time 1 moderated the relationship between work demands at Time 1 and burnout at Time 2 but did not moderate the relationship between work demands at Time 1 and mental health strain at Time 2.

### 3.3 Ancillary Analyses

Additional analyses were conducted to examine demographic differences on the primary variables. There were no significant differences on any of the variables as a function of gender (all *p*'s > 0.26). The effects of role (physicians, nurses, APPs) on stressors was marginally significant, *F*(2, 177) = 2.88, *p* = 0.059. Physicians (*M* = 3.90) reported marginally lower stressors than nurses (*M* = 4.75), *p* < 0.05. There were no significant effects of role on meaningful work. There was a significant effect of role on mental health strain *F*(2, 177) = 5.37, *p* < 0.05. Nurses (*M* = 2.36) reported more mental health strain than physicians (*M* = 1.47). There was also a significant effect of role on burnout *F*(2, 177) = 4.18, *p* = 0.017. Nurses (*M* = 2.43) reported higher burnout than physicians (*M* = 2.07).

## 4 Discussion

The present study examined how work demands and meaningful work are related to clinician burnout and mental health strain within the Delta surge of the COVID‐19 pandemic. The results of the study revealed that work demands at Time 1 were significantly correlated with burnout and mental health strain at Time 2, supporting Hypothesis 1, and that levels of meaningful work at Time 1 were negatively correlated with burnout and mental health strain at Time 2, supporting Hypothesis 2. In relation to Hypothesis 3, meaningful work at Time 1 was shown to moderate the relationship between work demands at Time 1 and burnout at Time 2. Contrary to our expectations, meaningful work did not moderate the relationship between work demands at Time 1 and mental health strain at Time 2. This could be due to strain being related to chronic mental health conditions, such as depression and anxiety, which can arise from multiple facets of life. In contrast, burnout is more a function of work‐related factors (Maslach and Leiter 2016).

The results of the present study are consistent with previous research showing relationships between work demands and burnout among EMCs. The connection between different types of work demands and negative outcomes in the present study are consistent with research by Ilić et al. (2017). These authors identified multiple psychosocial risk factors in the emergency department that resulted from demanding cognitive and emotional responsibilities, along with the high‐risk nature of needing to make quick, often life‐or‐death decisions.

The findings of the present study showing that meaningful work predicted burnout and mental health strain, as well as buffered the negative effects of work demands on burnout, are consistent with prior research highlighting the importance of meaningful work among healthcare professionals. These findings support meaningful work as an important job resource for mitigating the relationship between job demands and burnout for EMC (Bakker and Demerouti 2017). Ben‐Itzhak et al. (2015) revealed the importance of meaningful work in qualitative interviews with emergency physicians. Interestingly, Ilić et al. (2017) found that meaningful work was higher among physicians when compared to nurses, but the present study did not show significant differences in meaningfulness of work as a function of role. The present study adds to the findings of how meaningful work can help to alleviate the impact of work demands on burnout for different types of healthcare providers.

Although the present took place in the context of the COVID‐19 pandemic, the findings are likely to be generalisable beyond the COVID pandemic to other periods of high and sustained stress. EMCs are exposed to periods of sustained stress on a somewhat regular basis, including mass casualty situations involving natural or manmade disasters (Wrenn et al. 2010). Emergency Medicine (and healthcare in general) is hard and getting harder. Given the increased demands that EMCs face, meaningful work, and its buffering effects against work demands, is likely to be even more important moving forward.

### 4.1 Applications of the Findings

The present study adds to research in emergency medicine, addressing different work demands, meaningful work, burnout, and mental health strain in one study. Although emergency medicine is a critical specialty in the healthcare field, the Association of American Medical Colleges reports a significant shortage of clinicians, especially within emergency medicine. The COVID‐19 pandemic exacerbated these challenges, making it essential to understand the reasons behind clinicians leaving the field to address this issue (Heiser 2020). Furthermore, although the COVID‐19 pandemic has abated, burnout and turnover in the emergency medicine specialty continue to be elevated (McKenna 2024). We note that practically, there are two likely solutions to address these issues in emergency medicine: first, reduce work demands whenever possible, and second, intentionally increase the opportunities for meaningful work moments. Identifying factors that affect the risk of burnout and mental health strain should be recognized by those working in healthcare and their leaders in order to develop a healthier work atmosphere.

Given the importance of meaningful work in the present study, interventions that promote meaningful work as protection against burnout need to be developed and tested. Park (2013) has argued that successful meaning‐making can lead to stress‐related growth, thereby increasing job satisfaction and personal insight. Additionally, when healthcare professionals have a purposeful sense of meaning in their work, they may experience lower levels of burnout, as supported by Ben‐Itzhak et al. (2015). Potential interventions to increase meaningful work among EMCs include conveying patient feedback regarding benefits of treatment, reducing time spent on administrative tasks that detract from doing core treatment tasks, and recognition of important work by department and hospital leaders.

### 4.2 Limitations and Directions for Future Research

Although the COVID‐19 pandemic affected healthcare professionals over a period of years, the timeframe from this study took place over a two‐month period during the pandemic. Examining work demands and meaningful work as predictors of negative outcomes during this specific time frame leaves open the possibility that different time periods during the pandemic could influence the results that were obtained. For example, the start of COVID‐19 might have resulted in different work stressors and the sudden newness of the pandemic could have had a greater influence on burnout and mental health strain. These stressors could also vary in level of severity, depending on the time point within the pandemic, such as time points in which PPE was limited. Further research using data from other time frames could provide a broader overlook of the pandemic.

A second limitation of the present study involved the measure of job demands. The work demands that were generated were deemed relevant to the different roles examined in the present study. Furthermore, the demands examined were not generally different from one shift to another, although there are occasional differences overnight when fewer services are available. The present study was focused on the work demands present during shifts as opposed to shiftwork itself as a work demand. A more comprehensive set of job demands would include those related to shiftwork and an examination of differences in specific types of job demands based on the role of the clinician (e.g., nurses vs. physicians).

A third limitation of the study involves the relatively small number of participants for examining the job resource of meaningful work as a moderator of the job demands‐burnout relationship. Although our study used a longitudinal design, future research should recruit a larger sample of healthcare professionals and consider the use of mixed methods designs involving qualitative interviews to further examine how meaningful work reduces burnout among EMCs.

Finally, all variables and measures used in this study were self‐reported. Future research should consider using objective methods to assess burnout, mental‐health strain, and work demands. For example, De et al. (2003) conducted a study measuring the sympathetic‐adrenergic‐medullary axis and hypothalamic‐pituitary‐adrenal axis through salivary cortisol, heart rate, and blood pressure, showing participants who experienced burnout had higher resting heart rates and elevated cortisol levels compared to healthy controls.

## 5 Conclusion

The present study calls attention to the importance of understanding mental health strain and burnout within EMCs and working to find solutions to this prominent issue in healthcare. The findings highlight the relationships between work demands, burnout, and mental health strain, and that meaningful work can serve as a buffer against the negative effects of work demands on burnout. These findings have relevance to intervening to improve the health and well‐being of emergency clinicians.

## Funding

This research was supported in part by funding from the Clemson University School of Health, Prisma Health, and the National Science Foundation (#1654054, PI Shuffler). Any opinions, findings, and conclusions or recommendations expressed in this material are those of the author(s) and do not necessarily reflect the views of the funding agencies.

## Ethics Statement

Ethical approval has been granted by Prisma Health System IRB, Pro00058516.

## Conflicts of Interest

The authors declare no conflicts of interest.

## Data Availability

The data that support the findings of this study are available on request from the corresponding author. The data are not publicly available due to privacy or ethical restrictions.
